# Dr. Lacy B. Darling

**Published:** 1918-05

**Authors:** 


**Dr. Lacy B. Darling,** Long Island College Hospital, 1893, died at his home in Palmyra, Feb. 19, age 54.

